# Toward autonomous robotic-assisted and microrobotic surgery

**DOI:** 10.1126/sciadv.aec4197

**Published:** 2026-07-01

**Authors:** Sungyun Yang, Byung Ha Kang, Hyeongho Min, Juan P. Wachs, Farshid Alambeigi, Jaydev P. Desai, Pierre E. Dupont, Kurt Yaeger, Katherine Anderson, Alan Kuntz, Ron Alterovitz, Robert J. Webster, Nobuhiko Hata, Janani S. Reisenauer, McKenna Clinch, Alex Abramson, Jie Ying Wu, Tommaso Ranzani, Peter C. Kim, Alex S. Huang, Cheng Sun, Hao F. Zhang, Cameron N. Riviere, Eric R. Henderson, Michael Yip, Xiaoguang Dong, Daniel A. Wollin, David H. Gracias, Lamar O. Mair, Michael Karpelson, Robert J. Wood, Itai Cohen, Michael S. Strano

**Affiliations:** ^1^ Department of Chemical Engineering, Massachusetts Institute of Technology, Cambridge, MA 02139, USA.; ^2^ Edwardson School of Industrial Engineering, Purdue University, West Lafayette, IN 47907, USA.; ^3^ Walker Department of Mechanical Engineering and Texas Robotics, The University of Texas at Austin, Austin, TX 78712, USA.; ^4^ Wallace H. Coulter Department of Biomedical Engineering, Georgia Institute of Technology, Atlanta, GA 30332, USA.; ^5^ Department of Cardiac Surgery, Boston Children’s Hospital, Harvard Medical School, Boston, MA 02115, USA.; ^6^ Department of Neurological Surgery, Houston Methodist Hospital, Houston, TX 77030, USA.; ^7^ 1955 Capital & K2A2 Consulting LLC (Biomedical Engineering), Signal Mountain, TN 37377, USA.; ^8^ Department of Computer Science, Vanderbilt University, Nashville, TN 37212, USA.; ^9^ Department of Computer Science, University of North Carolina at Chapel Hill, Chapel Hill, NC 27599, USA.; ^10^ Department of Mechanical Engineering, Vanderbilt University, Nashville, TN 37240, USA.; ^11^ National Center for Image Guided Therapy, Department of Radiology, Brigham and Women’s Hospital and Harvard Medical School, 75 Francis Street, Boston, MA 02115, USA.; ^12^ Division of Thoracic Surgery, Department of Surgery, Mayo Clinic, Rochester, MN 55905, USA.; ^13^ School of Chemical and Biomolecular Engineering, Georgia Institute of Technology, Atlanta, GA 30332, USA.; ^14^ Division of Digestive Diseases, Emory University School of Medicine, Atlanta, GA 30307, USA.; ^15^ Department of Mechanical Engineering, Boston University, Boston, MA 02215, USA.; ^16^ Department of Surgery, Jacobs School of Medicine and Biomedical Sciences, University at Buffalo, Buffalo, NY 14203, USA.; ^17^ Activ Surgical Inc., Boston, MA 02210, USA.; ^18^ Hamilton Glaucoma Center, Viterbi Family Department of Ophthalmology and the Shiley Eye Institute, University of California San Diego, La Jolla, CA 92093, USA.; ^19^ Department of Mechanical Engineering, Northwestern University, Evanston, IL 60208, USA.; ^20^ Department of Biomedical Engineering, Northwestern University, Evanston, IL 60208, USA.; ^21^ Robotics Institute, Carnegie Mellon University, Pittsburgh, PA 15213, USA.; ^22^ Geisel School of Medicine, Dartmouth College, Hanover, NH 03755, USA.; ^23^ Department of Electrical and Computer Engineering, University of California San Diego, La Jolla, CA 92093, USA.; ^24^ Department of Biomedical Engineering, Vanderbilt University, Nashville, TN 37212, USA.; ^25^ Vanderbilt Institute for Surgery and Engineering, Vanderbilt University, Nashville, TN 37212, USA.; ^26^ Department of Urology, Brigham and Women’s Hospital, Boston, MA 02115, USA.; ^27^ Department of Chemical and Biomolecular Engineering, Johns Hopkins University, Baltimore, MD 21218 USA.; ^28^ Department of Chemistry, Johns Hopkins University, Baltimore, MD 21218, USA.; ^29^ Department of Materials Science and Engineering, Johns Hopkins University, Baltimore, MD 21218, USA.; ^30^ Department of Oncology, Johns Hopkins University School of Medicine, Baltimore, MD 21205, USA.; ^31^ Weinberg Medical Physics Inc., North Bethesda, MD 20852, USA.; ^32^ Harvard John A. Paulson School of Engineering and Applied Science, Harvard University, Cambridge, MA 02139, USA.; ^33^ Laboratory of Atomic and Solid-State Physics, Cornell University, Ithaca, NY 14850, USA.; ^34^ Kavli Institute at Cornell for Nanoscale Science, Cornell University, Ithaca, NY 14850, USA.

## Abstract

Autonomous robotic-assisted surgery (RAS) has emerged as a promising objective in biomedical technology, further enhanced by miniaturization toward microrobotic-assisted surgery (μ-RAS). This reduction in scale promises minimally invasive, partially or fully automated surgical procedures, with the potential to reduce patient recovery times, lower medical costs, and enable previously unavailable procedural options. This perspective highlights the specific advances in RAS that potentially map to the microscale (μ-RAS), organized across five surgical domains: endovascular, endoluminal, laparoscopic, ophthalmic, and orthopedic. We examine both clinical demands and technological advances in surgical robotics and identify the key innovations required for progress across these surgical fields. Our contribution is distinct in combining the perspectives of both surgical experts and bioengineering innovators, outlining a roadmap for the advancement and eventual integration of autonomous RAS and μ-RAS into mainstream surgical practice.

## Teaser

Robotic and microrobotic surgery is advancing across endovascular, endoluminal, laparoscopic, ophthalmic, and orthopedic care.

## INTRODUCTION

Since the first robotic surgery was developed in 1985 to perform neurosurgical biopsies (*1*, *2*), robotic-assisted surgery (RAS) has become increasingly prevalent in clinical practice. Sheetz *et al.* (*3*) reported that the use of robotic approaches in general surgery increased from 1.8 to 15.1% between 2012 and 2018, reflecting both technological advances and growing clinical acceptance. In parallel with the evolution of RAS, the concept of medical microrobots emerged more than two decades ago (*4*, *5*), with microrobots for minimally invasive surgery (MIS) later discussed in works by Fernandes and Gracias (*6*) and Nelson *et al.* (*7*). Several advances (*8*, *9*) in the field of microrobotics bring the prospect of miniaturizing RAS systems closer to clinical reality. Microrobotic-assisted surgery (μ-RAS) holds the potential to radically transform surgical practice by introducing minimally invasive procedures, whereby the tools may enter even without surgical incision or tissue damage, but rather through an existing orifice. As microrobotic systems become partially or fully automated, the level of procedural sophistication can increase.

Our perspective and review on this topic is unique for several reasons. Among our authors, we assembled practicing surgical experts who focus on contemporary robotic surgery. Specifically, we divided this biomedical space into five areas of specialization and organized the perspective accordingly: endovascular, endoluminal, laparoscopic, ophthalmic, and orthopedic systems. Our author list also includes pioneers in the domain of microrobotic systems who have demonstrated cutting-edge capabilities with a particular emphasis on surgical applications. Most of the authors met in person on 10 June 2024 at a full-day workshop organized around this specific topic. This provided for a rare, in-person intellectual exchange that has informed the contents herein. We note that this is unique for academic perspectives of this type but comes at a slight detriment of biasing the authorship to primarily US-based scholars, for which we ask the reader’s indulgence. Our review does not cover the laboratory demonstrations of microrobotic capabilities that some of our authors have already summarized elsewhere (*9*). We direct the reader to several outstanding reviews on robotic surgery itself (*10*, *11*) and the use of artificial intelligence (AI) to assist RAS in general (*12*–*14*). There has also been substantial research effort in microrobotic drug delivery devices, and this is not covered in our surgical perspective/review, but we direct the reader to outstanding reviews by Liu *et al.* (*15*), Gao and Wang (*16*), and Zhang *et al.* (*17*) We are also distinct from the proposed roadmap on clinical microrobotics translation recently proposed by Sitti and co-workers (*18*) because we focus only on the surgical domain and emphasize that opportunities across each surgical area remain distinct, as informed by practicing experts in each specialty. A single robotic platform is unlikely to affect all surgical domains equally. Our approach is to start from the current robotic surgical state of the art and review how emerging microrobotic tools can advance each area. Both this perspective and the authors advancing it are unique in this interdisciplinary research space. In summary, this current review focuses specifically on the emerging field of RAS and μ-RAS, drawing on both surgical practice and engineering innovation to provide an integrated perspective.

### The definition of autonomy

Recent breakthroughs in AI and machine learning (ML) are now propelling RAS toward its next frontier: autonomy (*19*, *20*). Autonomous robotic surgery aspires to enable robots to perform surgical tasks with limited or even no direct human intervention. The vision encompasses a spectrum of applications, from systems that provide decision support and automate routine tasks to those that could perform complex procedures in emergency situations or in environments where expert surgeons are not immediately available. In addition, autonomous systems could support less-experienced surgeons and potentially enable procedures beyond the current limits of human accessibility or perception (*21*).

To systematically address this evolving landscape, Yang *et al.* (*22*) proposed a framework of six levels of autonomy for robotic surgery, ranging from level 0 (no autonomy) to level 5 (full autonomy). While the highest level of autonomy has not yet been achieved, several commercial systems already operate at lower levels, offering measurable benefits through robotic assistance and task-specific automation (*20*). Recent advances in AI and ML have enabled surgical robots to imitate realistic surgery without human assistance (*23*). The field has attracted notable attention, and the role of AI in robotic surgery has been highlighted in several recent reviews (*10*–*14*). However, AI-assisted autonomous RAS still largely exists in a similar form factor, primarily serving as an aid to clinicians without fundamentally changing clinical practice.

## RESULTS

### Autonomous robotic endovascular surgery

#### Current landscape in endovascular interventions

Endovascular procedures are well primed to benefit from autonomous robotics in multiple ways: There is an anatomical highway (arteries and veins) that can be imaged precisely, autonomously segmented by the computer, and used to plan navigation and treatment (*24*). All interventions are performed from within and constrained by this intravascular network. Furthermore, endovascular surgery is very simple, consisting of torquing and pulling or pushing a wire or catheter (*25*). Unlike other surgical techniques, these motions are mechanically replicable by a robot and are driven in response to visual feedback (fluoroscopy), rather than tactile feedback. For these reasons, endovascular surgery is already on the cusp of being automated and will likely make surgery safer, faster, and with less radiation exposure for the patient and surgeon.

Endovascular robotic systems with autonomous capabilities have potential to improve patient care in several distinct clinical scenarios. One example is that autonomous endovascular robotic systems may increase access to emergency procedures, such as stroke thrombectomy and percutaneous coronary interventions (PCIs), which are usually performed at large medical centers with appropriately trained, specialized clinicians (*26*). In the United States, ischemic stroke, caused by a blood clot occluding a major cerebral artery, affects more than 690,000 patients annually (*27*), and multiple randomized trials have proven the long-term benefits of mechanical thrombectomy (*28*). Although this procedure has become ubiquitous in many large academic hospitals and certain smaller facilities equipped for stroke thrombectomy, access to such facilities is limited in the rural areas. Patients are often required to be transported from the rural facility to a tertiary care center, which takes away valuable time, before treatment can be administered (*29*). A similar challenge exists for the estimated 965,000 annual US heart attacks involving complete coronary artery occlusion (*30*). While emergency transcatheter removal of the clot and stenting represent the best treatment, this procedural capability is typically only available at larger medical centers. By locating robotic catheter systems in local hospitals, integrating partial autonomy, and providing remote specialist supervision, endovascular interventions for stroke and cardiac emergencies could be delivered more swiftly and in a broader range of settings.

Another compelling scenario involves complex intervention performed by a relatively small number of highly trained clinicians, enabled by partially autonomous robotic catheters. These include transcatheter valve repair and replacement procedures, brain aneurysm treatments, and ablation procedures for cardiac arrhythmias. The recent development of transcatheter valve interventions has extended the lives of patients who are too old and frail for open heart surgery. Their success has extended their use to younger patients, enabling them to avoid the trauma and risks of surgery. However, these procedures demand substantial training and consistent case volume for practitioners to achieve and maintain proficiency. For example, in edge-to-edge repair of the mitral valve, an inflection point in procedure success is observed after 50 cases, and outcomes continue to improve at 200 cases (*31*). Robotic catheters that incorporate autonomous elements could enable less experienced operators to perform at a level closer to that of high-volume experts, making such complex interventions accessible to additional hospitals.

Clinical and translational studies support the feasibility and safety of robotic-assisted endovascular intervention, particularly in coronary procedures. Telerobotic PCI with the operator and patient in different locations has been demonstrated in limited clinical experience, including a first-in-human study with the operator located ~20 miles away (*32*), and longer-distance operation has been evaluated in a transatlantic preclinical feasibility study of coronary angiography (*33*). Robotic-assisted PCI has advanced further into clinical evaluation, with prospective multicenter studies demonstrating feasibility and procedural safety (*34*, *35*). By comparison, robotic stroke thrombectomy remains at an earlier stage of development. For example, magnetic control approaches for neurovascular navigation are now in development and undergoing preclinical validation rather than routine clinical adoption (*36*).

**Fig. 1.**
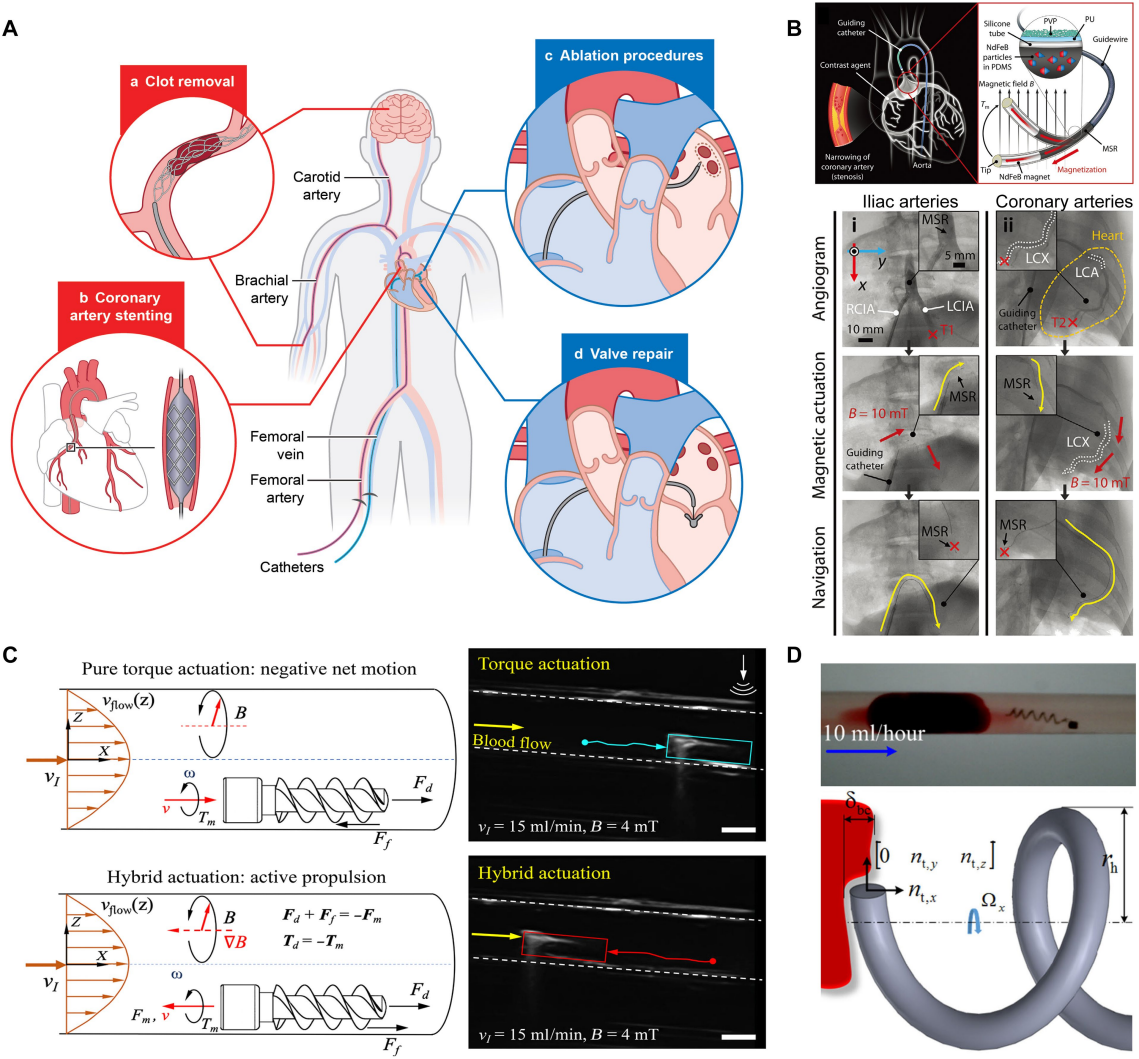
Autonomous robotic- and microrobotic-assisted endovascular intervention. (**A**) Schematic illustration highlighting the potential automation in endovascular surgery includes clot removal, coronary artery stenting, ablation procedure, and valve repair. (**B**) Schematics and experimental demonstration of electromagnetically controllable microrobotic intervention system for cardiovascular intervention. T_m_, magnetic torque. PVP, polyvinylpyrrolidone; PU, polyurethane; MSR, magnetically steerable robot; PDMS, polydimethylsiloxane; LCA, left coronary artery; LCX, left circumflex artery; RCIA, right common iliac artery. Adapted from (*42*) with permission from John Wiley & Sons. 2022 Wiley. (**C**) Ultrasound powered helical microrobot for accelerating thrombolysis in blood flow. B denotes the rotating magnetic field; T_m_, magnetic torque; T_d_, drag torque; v_flow_, flow velocity; v_I_, input flow rate; F_d_, drag force; F_f_, friction force; F_m_, magnetic force; and ω, angular velocity of the helical robot. Adapted with permission from (*43*). Copyright 2022 American Chemical Society. (**D**) Magnetic powered helical microrobot for blood clot removal. n_t,x_, n_t,y_, and n_t,z_ denote the x-, y-, and z-components, respectively, of the surface normal at the rotating tip of the tail in the robot frame, with n_t,x_ indicating the direction of rubbing against the clot; r_h_, radius of the helix; δ_bc_, incremental change in the volume of the clot; and Ω_x_, rotational frequency of the robot about the x axis. Reprinted with permission from (*44*). Copyright 2017 IEEE.

Over the past two decades, several robotic catheters designed for vascular navigation and cardiac ablation have been introduced clinically. For example, the Stereotaxis magnetic catheter system, initially developed for neurovascular navigation, now focuses on cardiac ablation (*37*), whereas Siemens Corindus system, first developed for coronary artery navigation, is now focusing on neurovascular navigation (*38*). Additional companies, some no longer operational, have introduced robotic systems for cardiac ablation (*37*). Despite these developments, widespread adoption has been limited because existing platforms often operate as teleoperated versions of conventional catheters, which improve operator ergonomics but add expense and procedure time without clear benefits for patient outcomes (*39*). To deliver substantial advantages in time critical or complex procedures, robotic catheters must expand their capabilities in both hardware design and algorithmic control, for treating aforementioned procedures, ranging from clot removal and coronary artery stenting to ablation procedures and valve repairs (Fig. 1A).

The first robotic technology challenge is the development of improved guidewire and catheter technology to provide reliable steerability, compliance control, and stability when navigating through tortuous vasculature and when manipulating tissue inside the blood vessel or organ. A second technological challenge involves the development of secure, high-bandwidth data transfer to support remote telesurgery and telementoring in time-critical interventions. These improvements must be achieved without substantial increases in learning curve or costs. Now, only a few companies are pursuing such innovations. Telos Health (Imperative Care) aims to broaden access to stroke care with its emerging robotic system, whereas Capstan Medical recently announced the successful completion of robotic catheter-based mitral valve replacements (*40*). Both systems remain in early development and are not yet commercially available.

#### Future opportunities with automation in endovascular surgery

Progress in AI and automation is required in two main areas. First, AI-assisted planning and guidance systems must integrate multimodal imaging and procedure-based knowledge models to provide clinical operators, both on-site and remote, with patient-specific procedural coaching, teleproctoring, and augmented reality (AR) visualization. For example, to perform neurovascular clot retrieval, such a system could assist in identifying the location of the clot and map an optimal route through the vasculature for reaching the clot. The system would need to consider/be compatible with wire torque and appropriate catheter size options to provide the maximum stability during vascular navigation. Similarly, in arrhythmia treatment, the system could interpret cardiac maps and propose the most appropriate locations for targeted ablation.

Second, automated motion planning and navigation controllers must be developed to decompose endovascular procedures into manageable subtasks. Such technology would propose a subtask plan to the clinician for their review and approval. The system would then carry out the plan under close physician supervision. In the case of a remote expert operator, the system would include safety features enabling it to pause, if a fault was detected. The local staff would also need to be trained on how and when to intervene. The first systems to enter this space should adopt a level-up approach, enhancing the abilities of local staff while ensuring that errors do not lead to major complications.

To maximize benefits and minimize costs, there is minimal need to completely automate a procedure. Automation should be applied first to most challenging subtasks. For example, emergency mechanical thrombectomy is comprised of vascular access, navigation to the aortic arch, navigation from the arch to the intracranial vasculature, and then clot removal. The vascular navigation subtasks are the most challenging parts of the procedure and should be the primary targets of automation. Clot removal is less challenging and could potentially be automated or could be performed by local personnel under remote expert guidance. Vascular access is the easiest subtask and need not be automated because it can be performed well by the local staff with modest training. Other examples of difficult subtasks worthy of automation include device navigation, positioning and deployment in heart valve repair and creating closed curves of ablation lesions in arrhythmia treatment. Another use of automation is to reduce the cognitive load or stress during complications by having the robot autonomously take over simpler tasks (e.g., blood suction) and freeing the surgeon to focus on the more cognitively demanding tasks (*41*).

Alternatively, miniaturized microrobotic surgery platforms hold promise for expanding the scope of endovascular interventions. Hwang *et al.* (*42*) introduced an electromagnetically controllable microrobotic intervention system capable of precisely guiding micro diameter guidewires under real-time x-ray imaging, thereby enabling targeted interventions with minimized radiation exposure (Fig. 1B). Wang *et al.* (*43*) developed a millimeter-scale helical robot guided by ultrasound Doppler imaging in combination with a magnetic torque-force hybrid actuation, allowing it to navigate both upstream and downstream in dynamic blood flow (Fig. 1C). This approach enhanced thrombolysis efficiency by up to 4.8-fold compared to traditional tissue plasminogen activator techniques. Khalil *et al.* (*44*) further demonstrated that mechanical rubbing of magnetic helical robots can triple clot-removal speed compared to conventional chemical lysis (Fig. 1D). Although these μ-RAS techniques remain in an early phase of development, they illustrate the future potential for untethered, fully automated endovascular interventions.

#### The endovascular surgeon’s perspective

Automating key elements of endovascular procedures may be closer at hand than many anticipate. While new technology is needed to achieve its full potential, incremental autonomous functionality can be implemented very quickly, much as driver assistance features have been integrated into automobiles without requiring a complete redesign. Approaches that align with current workflows will likely be more readily accepted by clinical teams, whereas radical shifts demanding extensive retraining may encounter resistance.

One barrier to adopting autonomous endovascular functionalities is the higher cost and increased perceived risk compared to teleoperated robotic control. This has resulted in the business models of clinical endovascular robots being based on demonstrating benefits using teleoperated control. Clinical experience to date, however, suggests that teleoperation in itself is not sufficient to justify the costs of an endovascular robot; some level of autonomy is needed. Autonomous robotic endovascular systems must have demonstrable benefits through clinical outcomes to justify the cost. This can be achieved by improving access to procedures, which would directly result in improved patient outcomes and enhancing safety of individual procedures by improving navigation and procedural efficiency.

### Autonomous robotic endoluminal surgery

#### Current landscape in endoluminal surgery

Endoluminal procedures require navigating complex, tortuous anatomical pathways, such as lumens, deep within the body to perform diagnostic or therapeutic interventions. The current manual tools used in such endoluminal procedures can be counterintuitive to control. For instance, interventional pulmonologists and gastroenterologists must perform complex motions of rotating, translating, and applying lateral forces at the base of the endoscope while simultaneously actuating levers or knobs to bend the scope’s tip, which requires practitioners to spend substantial amount of time becoming experts in the manual control of flexible endoscopes. They must frequently adopt awkward hand positions and body postures, all while viewing a camera feed that moves and rotates in real time for navigation. As a result of the physical difficulty of these procedures, endoscopists experience high rate of musculoskeletal or ergonomic injuries, with survey-based studies indicating rates of injury or pain in 37 to 95% of practitioners (*45*, *46*).

In response to these challenges, commercial medical robot systems have emerged to assist endoscopists in navigating the endoscopes, holding the potential to aid these physicians. Robotic platforms such as Johnson & Johnson’s Monarch (*47*) and Intuitive Surgical’s Ion robot (*48*) have been introduced relatively recently with regulatory approvals in bronchoscopy and urology. These systems replace manual endoscopes with teleoperated robotic scopes, featuring ergonomic user interfaces that reduce the physical burden on clinicians and possibly offer more intuitive scope control. Although the Monarch and Ion systems have demonstrated the ability to mechatronically automate the mechanical control and motion of scopes for navigation, much of the navigation strategy and nearly all clinical decision-making still remains with the human operator. Even yet, robotic endoscopy has the potential to improve patient outcomes (*49*, *50*). This promise of potentially enhanced procedure accuracy and effectiveness, the difficulty of manual procedures, combined with the need to reduce injury rates among clinicians and the demonstrated feasibility of robotic teleoperation, makes autonomy a particularly promising direction for endoluminal interventions.

An endoluminal procedure generally involves three major aspects: navigating to a predetermined anatomical site, performing exploratory navigation of the lumen, and carrying out the actual intervention. Each of these components presents distinct challenges and opportunities for autonomy.

Navigation to a known anatomical site, such as a location deep in the bronchial tree near a suspicious lung nodule or a specific location in the urinary tract, may offer the greatest potential for autonomy in endoluminal procedures in the shorter term. If the anatomical site the robot must navigate to is known in advance, given the existence of the mechatronic systems described above, automation of this navigation becomes conceptually similar to automation in other robotics domains, where techniques like optimal control, motion planning, and computer vision can be applied and, in some cases, can provide provable performance guarantees.

A distinguishing characteristic of endoluminal procedures is that the lumen itself often constrains possible paths, effectively forming a conceptual two-lane route that reduces navigational complexity. Even the lumens with more complex structures, such as the tree-like topology of the airway, often lend themselves to straightforward path planning. Intuitively speaking, this takes much of the “guess work” out of large portions of the navigation task. As demonstrated by the Ion system (*48*), a robot can automatically map a route through the bronchial tree and display it to the clinician. Nonetheless, this does not entirely solve the problem as lumens such as the colon can deform substantially during procedures thereby complicating navigational autonomy. Moreover, fine positioning near the target becomes a less structured task, requiring precise alignment with dynamic local anatomy.

Exploratory navigation, in which the robot traverses the lumen to gather diagnostic or procedural information, represents an even more demanding scenario for autonomy. Unlike targeted navigation, where clinical decisions have typically been made beforehand, exploratory procedures require the system to search for lesions or anomalies, interpret sensor data and imagery, and incorporate knowledge of patient history or risk factors. Exploratory navigation requires the most medical reasoning capabilities, where it must reason about what it is seeing/sensing, how this relates to the patient’s history, presentation, risk factors, etc., to potentially then make interventional decisions. This is distinct from the aspects discussed above in which much of the clinical decision-making had already been made by human experts.

The third aspect is the intervention itself. Once the robot has reached the desired location, it must carry out a specific task, such as obtaining a biopsy from a lung nodule or retrieving a kidney stone. The short-term feasibility of automating the intervention after navigation heavily depends on the specific intervention. However, this is the aspect of one of the most advanced demonstrations of autonomy in endoluminal interventions in an academic setting. One advanced demonstration in this area showed automated peripheral lung nodule access using a steerable needle deployed through a bronchoscope, achieving clinically relevant accuracy in vivo and superhuman performance in ex vivo studies (*51*).

#### Future opportunities with automation in endoluminal surgery

As robotic hardware continues to improve, endoluminal systems will likely perform more complex tasks deep within the body. Systems capable of bimanual, dexterous manipulation within small-diameter lumens, or even untethered robots capable of independently navigating through the body, are increasingly within reach. Recent advancements in miniaturized microrobotic surgery have also been applied to endoluminal surgery, particularly within the gastrointestinal (GI) and urinary systems.

**Fig. 2.**
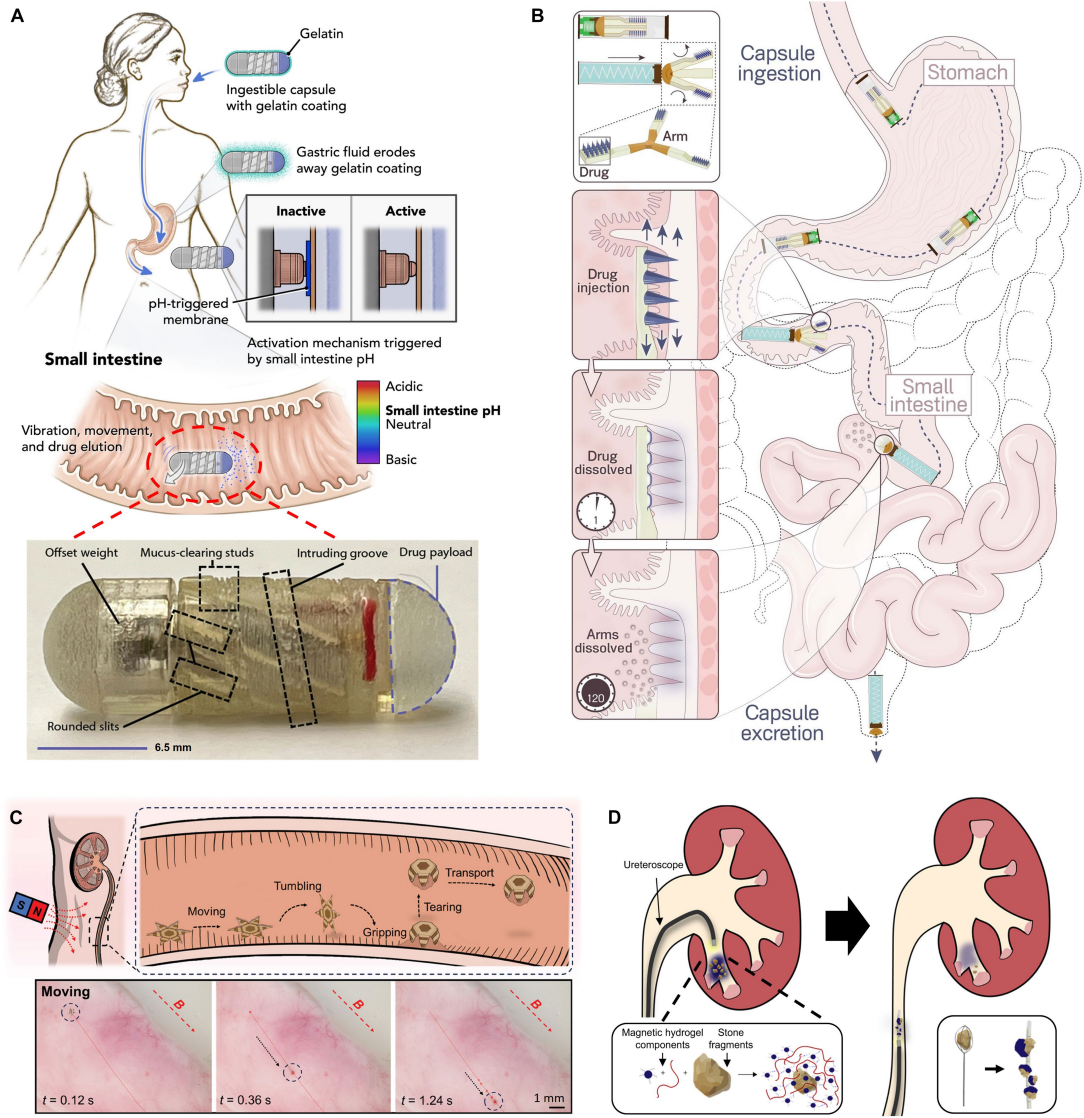
Autonomous robotic- and microrobotic-assisted endoluminal intervention. (**A**) Ingestion and activation processes of the ingestible smart pill for enhanced drug delivery in the gastrointestinal (GI) tract. Reproduced from (*52*), S. S. Srinivasan *et al.*, *Science Robotics*, DOI: 10.1126/scirobotics.abp9066 (2022), AAAS. (**B**) Activation process from ingestion to excretion of luminal unfolding microneedle injector pills actuated and unfolded in the small intestine, injecting drug loaded microneedles into the tissue wall. Reproduced from (*64*). (**C**) Schematic of a microgripper in the upper urinary tract under magnet control and series images of a microgripper move on ex vivo pig urothelial tissue. Reproduced from (*92*) with permission from John Wiley & Sons. 2024 Wiley. (**D**) Magnetic hydrogel components are introduced through the working channel of a ureteroscope to coat stone fragments in a superparamagnetic hydrogel (left). A magnetic wire is introduced to capture the magnetically labeled stone fragments. The ureteroscope and wire, along with the captured fragments, are removed from the body (right). Reproduced from (*93*), licensed under the Creative Commons CC BY license.

In the GI tract, one of the most representative approaches involves smart pills, which are orally ingested and travel through the digestive tract (*52*, *53*). Regional identifiers, such as pH (*54*–*56*), temperature (*56*), physiological biomarkers (*57*), oxidation reduction potential (*58*), pressure (*56*, *59*), and visual cues (*60*), have already been leveraged in the clinic to create sensing devices that can be used for autonomous actuation of diagnostic and therapeutic events. Preclinical capsules present the promise of pills that can detect x-ray radiation as well (*61*). Current clinical devices enable autonomous endoscopy (*60*) or mechanical stimulation (*62*), whereas preclinical devices have been shown to enable precise drug delivery (*63*, *64*) or electrical stimulation (*65*) (Fig. 2). Some devices use prestored energy in the form of compressed gas (*66*), compressed springs (*63*, *64*), and triggerable chemical reactions (*67*, *68*), whereas others have harvested energy from gut fluids (*69*) or used external magnetic manipulation (*70*, *71*). Random capsule orientation is a major hindrance to achieving predictable tissue contact in the stomach, which was successfully overcome by a self-orienting system engineered by Abramson *et al.* (*63*, *72*, *73*) in a pill used for the oral delivery of macromolecular drugs. Preclinical deformable materials (*74*, *75*) and folding mechanisms (*64*, *76*), as illustrated in Fig. 2B, are also leveraged for maximizing tissue contact and achieving device localization within specific organs.

The use of microneedles (*72*, *73*) and jetting systems (*66*, *77*) can enhance therapeutic drug delivery and electrical stimulation efficacy by bypassing thick mucus layers, as demonstrated preclinically. Some smart pills are designed with fluid wicking mechanisms (*52*, *65*), clearing the layer of mucus at the delivery site in swine. Ramadi *et al.* (*65*) developed a fluid-wicking, stomach-localizing, electrical stimulation capsule that modulated hormone production and triggered an increase in ghrelin plasma levels, stimulating feelings of hunger. Electrical stimulation of the GI tract has also been shown to increase the production of cholecystokinin (*78*) and glucagon-like peptide-1 (GLP-1) (*79*), where the former determines feelings of satiety and the latter stimulates insulin secretion. In addition to hormone secretion, electrical stimulation also induces peristalsis. Using conductive microneedles in the self-orienting capsule design, Abramson *et al.* (*80*) induced muscle contractions within the swine stomachs to promote digestion, and other smart fibers have been demonstrated to induce and measure peristalsis in the small intestine as well as measure serotonin gut levels, a neurotransmitter associated with peristalsis (*81*, *82*). Such approaches leverage the natural luminal pathways of the GI tract, minimizing surgical invasiveness while improving targeted treatment delivery.

Future innovations in automated endoluminal surgery will use advances in microelectronics and AI to reduce device size and improve targeted localization (*83*, *84*). Now, the large, nondegradable electrical and mechanical components required for ingestible devices hinder capsule passage and increase the risk of intestinal obstructions. The first generation of capsule endoscopy cameras, measuring 11 mm by 26 mm, was associated with a pooled intestinal obstruction rate of 1.4% in patients, whereas patients suffering from Crohn’s disease had a 13% obstruction rate (*85*, *86*). One way to reduce capsule size is to design custom application-specific integrated circuits, but this requires years of effort that must be redone for each application (*87*). Healy *et al.* (*88*) recently took a different approach, using commercial electronic components to design capsules that separated into tiny pieces to facilitate GI transit in swine. Delivering therapeutics to targeted areas also remains a challenging but important goal. Electrical stimulation therapies generate different hormones based on their location in the GI tract (*65*). Drugs that treat inflammatory bowel disease would benefit from localization to the inflamed area by reducing off-target side effects at lower dosage levels (*89*). Emerging localization methods may move beyond pH sensors to more complex chemical sensing mechanisms that detect local signatures of disease, such as localized hypoxia or cytokine up-regulation (*90*, *91*). This requires the development of robust sensors that can survive the harsh environment of the GI tract during transit.

For the urinary system, Liu *et al.* (*92*) have suggested that untethered microgrippers can be used for biopsy in the upper urinary tract. Microgrippers autonomously triggered by physiological temperature are maneuvered using an external magnetic field (Fig. 2C). The biopsied tissue samples were successfully collected from ex vivo pig ureters. Ge *et al.* (*93*) have introduced the efficient retrieval of kidney stone fragments during ureteroscopy. Magnetic hydrogel components are introduced through the ureteroscope and coated to stone fragments (Fig. 2D). Then, a magnetic wire is activated to capture the magnetically coated stone fragments and safely removed as the ureteroscope is retracted from the body. These recent advancements in miniaturized microrobotic surgery highlight the potential for improving the precision and efficacy of endoluminal interventions. However, many of these procedures still rely on manual operation, which presents substantial challenges in terms of procedural complexity and physician ergonomics.

Still, many challenges must be overcome before such visions can be realized. Manipulators need to provide sufficient strength and stiffness in tight spaces while retaining fine control. Deformable registration, scope shape sensing, and in situ tissue characterization must be improved to enhance the robot’s perception and situational awareness. Beyond executing a preplanned route, higher levels of autonomy will also require systems that can interpret findings during exploratory navigation and adjust procedural plans under deformable and dynamic anatomy. Furthermore, higher-level cognitive functions, including semantic scene understanding, task and skill planning, and adaptive learning, remain areas that require substantial development.

#### The endoluminal surgeon’s perspective

From the surgeon’s viewpoint, endoluminal procedures can be both mechanically demanding and cognitively intensive. High rates of musculoskeletal injury highlight the physical toll of manually manipulating flexible endoscopes, often in awkward positions or orientations. Even teleoperated robotic scopes, while reducing ergonomic strain, leave critical decisions about navigation, lesion identification, and interventional strategy to the clinician.

The potential for AI-driven autonomy to share this cognitive burden is especially appealing, whether by suggesting optimized routes through the anatomy or assisting with tasks such as lesion recognition and targeted biopsy. These opportunities raise vital questions related to patient safety, regulatory oversight, and clinician trust. Nevertheless, automation in endoluminal interventions aims to assist, rather than replace, clinical expertise. By reducing physical strain, streamlining navigation, and supporting real-time decision-making, autonomous robotic systems have the potential to make procedures safer and more effective for both patients and physicians.

### Autonomous robotic laparoscopic surgery

#### Current landscape in laparoscopic robotic surgery

MIS has steadily advanced surgical practices by minimizing tissue trauma while broadening surgical options to improve patient outcomes, shorten recovery times, and enhance long-term quality of life (*94*–*97*). Laparoscopy, a principal MIS technique, involves creating small incisions (0.5 to 1.5 cm) in the abdomen, which is then inflated with CO_2_ to provide an operative field (*98*). In the United States, more than 750,000 gallbladder removals, 600,000 hysterectomies, 300,000 bariatric procedures, 300,000 bowel resections, 250,000 appendectomies, 250,000 lung resections, and 90,000 prostate surgeries are performed annually, with a growing proportion now adopting an endoscopic or laparoscopic approach (*99*–*101*).

Endoscopic technology has transformed surgical visualization from a passive, human-eye analog system into a digitalized, interactive modality. This shift has introduced additional coregistered visual inputs, such as near-infrared imaging with fluorescent dyes, laser speckle imaging, and other multimodal sensors, often overlaid as AR features (*102*–*105*). Such real-time physiological and anatomical signals facilitate more precise decision-making and potentially improve patient outcomes. Further, recent applications of ML and multimodal large language models to endoscopic video are beginning to identify critical procedural steps and contextual cues, offering a glimpse of how future “copilot” AI systems might assist surgeons in perceiving and interpreting intraoperative events (*106*–*110*).

Despite these advancements, performing complex laparoscopic procedures remains challenging due to the long, rigid instruments with simple distal end effectors and minimal sensory feedback. Surgeons rely heavily on visual cues, risking tissue damage when handling large or fragile organs such as the liver or bowel (*111*–*114*). These limitations become particularly problematic for patients with organs in a nonphysiological state, where tissues may be less resilient to applied forces. Moreover, procedures often require multiple assistants for organ retraction or camera manipulation, driving up personnel needs and associated costs. Consequently, laparoscopic adoption rates remain lower in resource-limited settings, as seen in rural areas where patients are substantially less likely to receive laparoscopic colectomies for colon cancer (*115*, *116*).

Various hardware solutions have emerged to reduce the number of required incisions and skilled personnel. Magnetic systems and mucosal adhesive devices, introduced through small incisions or natural orifices, can anchor internal instruments within the abdominal cavity, thereby expanding the operative field beyond the constraints of standard trocars (*117*–*122*). However, these methods face reliability issues, such as potential loss of magnetic coupling, and are limited by patient-specific factors like abdominal wall thickness (*123*–*125*).

#### Future opportunities with automation in laparoscopic surgery

Robotic systems for laparoscopy have improved distal dexterity and ergonomics but largely remain teleoperated and offer low levels of autonomy (*20*, *126*). Commercially available multiport platforms such as the da Vinci Xi, Versius, and Senhance are often expensive and require extensive surgeon training, limiting their use to high-volume centers where costs can be offset. Handheld robotic devices address some infrastructure challenges but still lack comprehensive sensor feedback for force or tissue characterization, perpetuating the risk of tissue injury.

To advance toward autonomy, laparoscopic robotic systems need more robust anatomical modeling and three-dimensional (3D) scene understanding. Unlike orthopedic surgery, which benefits from rigid anatomy and optical trackers, soft tissues in laparoscopy are deformable and vary widely between patients. Now, the endoscope video serves as the primary sensor input, creating major challenges for real-time 3D reconstruction and safe manipulation (*127*, *128*). Autonomous control loops must incorporate compliance to accommodate tissue movement and reduce iatrogenic injuries. Improved anatomical modeling and 3D scene understanding are, therefore, expected to first enable low-level, task-specific autonomy for well-defined subtasks, such as stable camera positioning and related assistive tasks, rather than higher-level autonomous procedural decision-making (*129*). Higher-level autonomous decision-making remains challenging in deformable soft-tissue environments, so near-term progress will likely be achieved through incremental, surgeon-in-the-loop automation that executes approved subtasks under safety constraints (*130*).

Near-term steps toward automation could focus on augmenting perception with additional sensing modalities (e.g., advanced fluorescence imaging, spectroscopy, or depth sensing) that might not be directly visible to the surgeon but can be integrated into a computer-generated 3D model (*131*, *132*). ML algorithms can then learn from large datasets of surgical procedures, capturing how tissues deform under both normal conditions and surgical manipulation (*128*). Such data would also clarify how expert surgeons adapt to changing intraoperative conditions, providing a foundation for autonomous task execution.

Furthermore, digitalization in laparoscopy enables real-time data analysis and feedback, potentially improving situational awareness. Tools and endoscopes could be equipped with positional tracking, force sensing, and even haptic feedback to prevent excessive pressure on tissues (*133*). Soft robotic systems, such as tentacle-like or morphable instruments, could enhance maneuverability and reduce trauma by matching compliance with target tissues (*134*–*137*). By stepping away from purely anthropomorphic device designs, laparoscopic robots may ultimately achieve more efficient, tissue-specific manipulation.

#### The laparoscopic surgeon’s perspective

Despite technological advancements, the present generation of robotic laparoscopic platforms offers improved dexterity but remains limited by cost, training requirements, and inconsistent impact on clinical outcomes (*138*, *139*). For instance, robotic surgery has not notably reduced iatrogenic complications, which range from less than 1 to 10% in certain procedures, implying robotic assistance alone has not solved key challenges associated with tissue handling and visualization (*138*, *139*).

Global surgical needs further highlight where automation could have the most profound effect. According to the Lancet Commission, of the 310 million surgical procedures performed worldwide each year, ~143 million more are needed to address unmet healthcare demands, primarily in resource-constrained settings (*140*). High-end technology often has limited utility for bridging this gap unless costs, training barriers, and logistical demands can be substantially lowered.

Ultimately, building trust in autonomous or collaborative robotic solutions will require more than technical feasibility (*22*, *126*, *130*). Lessons from autonomous driving suggest that user acceptance depends not only on demonstrated safety but also on clear, incremental steps from lower-risk tasks to more complex procedures. Surgeons must see tangible benefits, such as reduced mental and physical strain, improved consistency, or fewer errors, for automated systems to gain traction. By derisking and validating these technologies at the level of simple, well-defined subtasks (e.g., controlled organ retraction or stable camera positioning), developers can establish a foundation of confidence. This measured progression offers a pathway toward a future in which autonomous systems and surgical teams collaboratively improve patient outcomes, reduce complication rates, and make minimally invasive procedures more accessible worldwide.

### Autonomous robotic ophthalmic surgery

#### Current landscape in ophthalmic robotic surgery

Ophthalmic surgery includes a wide range of procedures used to treat various ocular disorders. The most amenable to early automation, due to minimal requirements for direct physical contact, are laser therapies applicable to both the anterior segment (e.g., laser iridoplasty, iridotomy, and capsulotomy) and posterior segment (e.g., photocoagulation and photodynamic therapy). Subsequent targets include minor procedures now performed in an office setting, such as bleb revisions, diagnostic or therapeutic anterior chamber taps, and intravitreal drug or implant injections into the posterior segment. Last are intraocular surgeries performed in the operating room. These are shorter and typically involve fewer steps than in general surgery. Unique to ophthalmology, ophthalmic procedures often take place entirely inside of an organ, the eye, rather than on sequentially exposed tissue layers, and they benefit from the inherent optical transparency of the eye. This characteristic allows for direct visualization of most intraocular anatomy, facilitating both human and robotic assessment.

In the past two decades, refractive surgery has incorporated automation into certain aspects, such as microkeratome actuation in automated lamellar keratoplasty (*141*) and automated control of excimer lasers with tracking and compensation of eye movement in procedures such as laser in situ keratomileusis (*142*). Nonautonomous robotic-assisted vitreoretinal microsurgery has been demonstrated clinically, including retinal vein cannulation using a cooperatively controlled robot system (*143*) and membrane peeling and subretinal injections using a telerobotic system (*144*). In a first-in-human randomized trial of robotic-assisted epiretinal or inner limiting membrane peeling, surgical success was comparable and retinal microtrauma was not notably different from manual surgery, while the dissection time was longer with robotic assistance (4 min 55 s versus 1 min 20 s) (*144*). However, the robotic system substantially improved operation stability and accuracy. For context, examples of intraocular tissues that either need to be handled, peeled, or protected include the posterior lens capsule (~4 to 6 μm) (*145*), internal limiting membrane (ILM; 400 to 1400 nM) (*146*), and Descemet membrane endothelial keratoplasty grafts (10 to 15 μm) (*147*). Unaided human physiological tremor typically limits manual dynamic precision to 40 to 100 μm at the instrument tip. In contrast, reported robotic telemanipulation systems can filter this tremor to achieve 1- to 10-μm precision (*144*). Furthermore, recent studies indicate that robot-assisted membrane peeling reduces the required number of grasping maneuvers per minute and shows a strong trend toward fewer intraoperative microhemorrhages compared to manual techniques (*148*). Separately, a prospective phase 1 trial in four patients with central retinal vein occlusion demonstrated the feasibility of robotic-assisted retinal vein cannulation with intravascular ocriplasmin infusion (*149*). Numerous other robotic platforms for ophthalmic surgery are under development, and many remain preclinical.

Ophthalmic microsurgery is one of the most technically demanding specialties in terms of the requisite accuracy in manipulation, and this leads to considerable challenges in the development of robotic manipulators for such procedures. Recently, notable progress has been made in sensing and imaging modalities of the surrounding anatomic features at microscale resolution and accuracy [e.g., optical coherence tomography (OCT)], but further advances are needed to achieve high-speed 3D freeform imaging and to accurately integrate volumetric sensing/imaging results from multiple detection volumes into a cohesive digital twin. Such platforms would provide comprehensive intraoperative guidance and feedback control.

**Fig. 3.**
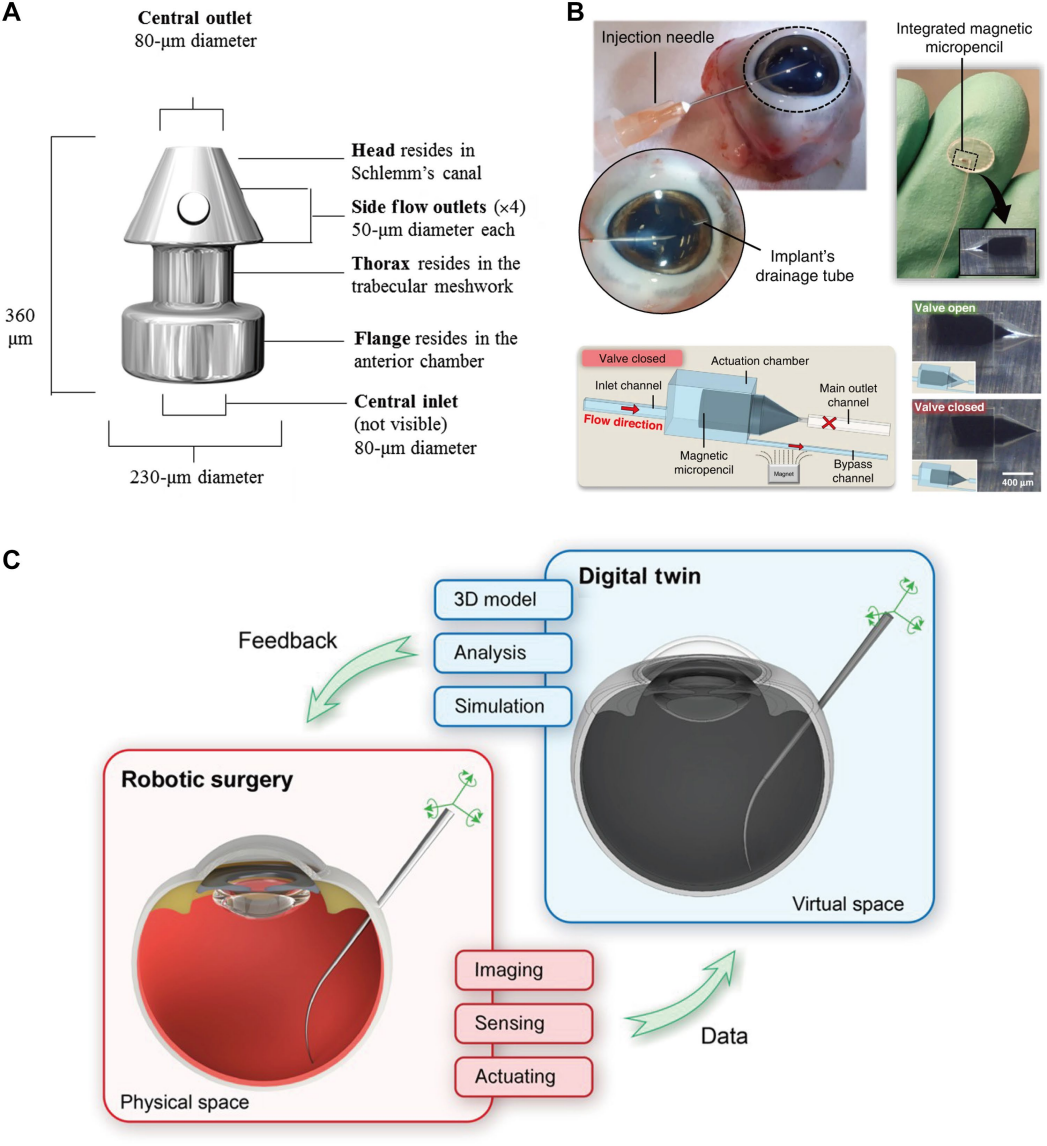
Autonomous robotic- and microrobotic-assisted ophthalmic surgery. (**A**) iStent inject GTS 400 (Glaukos Corporation, San Clemente, CA) stent design and dimensions. Reprinted from (*150*), Ophthalmology, 126, T. W. Samuelson *et al.*, Prospective, randomized, controlled pivotal trial of an ab interno implanted trabecular micro-bypass in primary open-angle glaucoma and cataract: Two-year results, pp. 811–821, Copyright (2019), with permission from Elsevier. (**B**) Illustration for overview of robotic microstent. Experimental setup used for the ex vivo experiments, fabricated magnetically actuated glaucoma implant with an integrated valve containing the micropencil, a 3D zoomed view of the microvalve (total length of 1 mm, largest diameter of 357 μm) demonstrating the actuation mechanism, and microscopic images showing the microvalve in both open and closed states. Reproduced from (*153*), licensed under the Creative Commons CC BY license. (**C**) Schematic illustration highlighting the synergy between a robotic surgery platform and its digital twin for ophthalmic interventions. In the physical space, the robotic system performs imaging, sensing, and actuation, generating data that are transmitted to the digital twin in the virtual space. The digital twin processes these data through 3D modeling, analysis, and simulation, enabling real-time feedback on surgical instrument positioning and motion dynamics. This feedback loop allows for precise control and actuation of curved surgical tools via high-degree-of-freedom robotic motions, enhancing surgical accuracy and improving patient outcomes.

One emerging technology complementary to such advanced imaging and control systems is the robotic stent (Fig. 3A) (*150*). In particular, the development of minimally invasive glaucoma surgery has increased demand for robotic solutions in ophthalmology, which already benefit cataract and retinal surgeries by enabling stable microsurgical manipulation and reproducible outcomes. For example, the US Food and Drug Administration–approved iStent injection has demonstrated efficacy in lowering intraocular pressure (IOP) while minimizing ocular tissue trauma (Fig. 3A) (*150*–*152*). However, real-world device malfunctions, such as inadvertent stent misplacement, highlight the need for further improvements in both robotic platforms and implantable ophthalmic devices. A recent study introduced a magnetically actuated glaucoma drainage device that leverages magnetic forces to control microvalves, thereby allowing noninvasive IOP regulation post-implantation (Fig. 3B) (*153*). This type of “smart” implant technology illustrates the growing potential of robotics to provide safer, more efficient treatment options for glaucoma patients and underscores the broader impact that robotics may have on the future of ophthalmic care.

#### Future opportunities with automation in ophthalmic surgery

The potential benefits of fully autonomous robotic ophthalmic surgery include the ability to perform urgent procedures when a surgeon is not available and to automate labor-intensive high-volume tasks, thereby reducing costs and standardizing outcomes. For instance, fully automated robotic laser treatments could be used for urgent laser peripheral iridotomies in acute angle closure crises or for common retinal photocoagulation in diabetic retinopathy, the prevalence of which is expected to increase (*154*). Minor yet critical procedures, such as emergency canthotomy and cantholysis for retrobulbar hemorrhages, as well as the widespread administration of intravitreal injections for macular degeneration, a disease affecting nearly 20 million Americans (*155*), could also be automated. Last, for surgery, autonomous robotic systems could be used for globe trauma repair to minimize the time between presentation and eye stabilization, as well as for routine cataract surgeries, which account for ~$3.4 billion in annual Medicare expenditures (*156*).

To reach both short-term and long-term autonomy goals, technological advancements are required across hardware, perception, and decision-making intelligence (Fig. 3C). One of the key areas of development includes designing curved robotic tools to provide access to all areas of the eye, an improvement over existing straight tool (*157*). These surgical tools should be quickly removable, offer additional degrees of freedom without requiring internal joints, and include specific instruments like curved forceps for tissue dissection, optical fibers for illumination or laser applications, and adjustable microneedles that can align with vessels behind the sclera. Such advancements would enable intricate surgical maneuvers and improve outcomes in complex procedures.

Visualization also requires substantial improvement, focusing on integrating OCT and digital microscope images to comprehensively visualize structures such as the iris, sclera, capsule, and retina (*158*, *159*). Real-time 3D reconstruction and visualization of the eye would enhance surgical navigation, improving accuracy and reducing risks. Additionally, force sensing and recognition of surgical signatures, such as tissue removal, acoustic emissions, color changes, and flow alterations, are essential for better understanding the surgical environment. The ultimate goal is to achieve “straight-ahead surgery,” reducing the need for unnatural eye movements and enhancing surgical precision and patient comfort.

An ideal autonomous surgical robot for ophthalmic procedures should provide improved access to all regions of the eye while simultaneously reducing costs. It must have highly precise manipulation capabilities with precision under 10 μm in 3 to 6 degrees of freedom and real-time submicrometer sensing. Improved OCT imaging with high resolution, fast acquisition rate, and optimal depth of field is essential. Quantitative evaluations of manual ILM peeling indicate that involuntary human force errors average around 0.50 mN (*160*). Therefore, future autonomous systems must not only maintain sub-10-μm spatial precision but also integrate high-fidelity force-sensing to remain well below the 0.50-mN threshold, thereby actively reducing the risk of iatrogenic retinal trauma (*149*, *160*, *161*). The robot’s sensor processing must interpret data streams and display information with a lag of no more than 80 ms. Additionally, the robot response time should be within 200 ms to close the loop, make decisions, and begin executing an action. Because most patients are only sedated during these procedures, the robot must be capable of withdrawing tools within 200 ms to ensure patient safety in case of unexpected movement. In the case of robotic stents, automated safety mechanisms would allow them to adjust or retract if improper placement or adverse physiological responses are detected, further enhancing patient safety.

#### The ophthalmic surgeon’s perspective

The challenge of fully autonomous procedures is less in the procedural action and more in evaluating the anatomical surroundings followed by decision-making. Imaging is necessary to generate information about the environment. AI is needed to integrate this information with the procedural action. Using an anatomical “digital twin” of patient’s eyeball for personalized surgical planning can also facilitate interoperative decision-making. To teach a robot, individual steps can be taught to simplify the overall training. However, requiring a surgeon to “transition” the robot between steps negates many of the benefits of full automation. Initially, stepwise actions will need to include teaching identification of the target tissue, static surgical steps, and then linking these steps to create a seamless and dynamic process. To achieve this, we estimate needing not only at least 1000 training cases but also the requirement to define measurable success measures to grow surgeon confidence. Last, a “stop” function must be developed to give the surgeon an opportunity to step in and take over. Monitoring for the “stop” could be performed by the robotic AI itself or by trained “medical extenders” [physician assistants (PAs) or nurse practitioners (NPs)] to avoid a 1:1 surgeon-patient ratio. As a starting point, we propose the following general steps to achieve fully autonomous ophthalmic surgery: (i) platform development and integration (workflow, combining modalities), (ii) visualization and imaging, (iii) real-time fusion of the digital microscope and OCT for the surgeon, (iv) active sensing and real-time feedback of surgical outcome, (v) implementation of digital twins, (vi) detection learning, (vii) autonomous modeling, and (viii) decision-making.

To enhance a surgical robot’s capabilities beyond vision and 3D perception, multiple information streams should be incorporated. A fused display of OCT and digital microscope images in real time would greatly assist both manual and assisted surgeries, reducing the need for surgeons to merge complex imagery from two separate screens. Haptic feedback would add tactile information, enhancing the surgeon’s ability to control delicate procedures. Additionally, the development of digital twins, or virtual replicas of the patient’s eye, would be instrumental for presurgical planning and real-time feedback, providing predictive models that guide the surgical process. Contextual awareness, such as monitoring anesthesia levels and tracking movement in the operating room, would further improve situational awareness, supporting safer and more effective surgical interventions.

### Autonomous robotic orthopedic surgery

#### Current landscape in orthopedic robotic surgery

Orthopedic surgery focuses on managing issues of the musculoskeletal system, ranging from stabilizing fractured bones to replacing arthritic joints and repairing damaged ligaments or tendons (*162*). In many of these procedures, skeletal anatomy provides a robust point of orientation, directly in cases involving bony pathology and indirectly when treating ligament or tendon injuries. Consequently, orthopedic surgery has benefitted from the advent of x-ray–derived imaging, mainly single-dimension fluoroscopy and computed axial tomography (CT) for navigation and guidance.

Compared to other surgical specialties, where nonrigid tissues pose greater challenges, orthopedics is particularly amenable to near-term advances in robotic autonomy. Procedures such as fracture reduction and implant placement already rely on interpreting fluoroscopic images and performing physical maneuvers to restore proper alignment. Much of this is rooted in basic geometric calculations, making it feasible to adopt assembly line-type robots, provided that real-time visualization of the anatomy is maintained. Beyond CT-based navigation and fluoroscopy, additional tissue-characterization methods (e.g., fluorescence probes for perfusion or enzyme activity, tissue impedance measurement, nerve monitoring, and oxygen saturation/perfusion monitoring) could further expand the capabilities of autonomous orthopedic surgeries. Accordingly, recent studies have demonstrated early feasibility of image-guided robotic execution in orthopedics, including cadaveric validation of planned drilling and cement injection for osteoporotic hip augmentation (*163*). Early clinical work has also reported robotic femoral fracture reduction using model-based planning, achieving improved alignment accuracy with fewer fluoroscopic images than conventional techniques (*164*).

#### Future opportunities with automation in orthopedic surgery

Although orthopedic surgery has substantially benefited from imaging technologies and semiautomated navigation, advanced AI-driven solutions offer opportunities for real-time decision-making, precision alignment, and fully or partially automated surgical execution. Identifying a specific first procedure for automation should consider several factors that justify the effort and facilitate success, including: (i) procedure commonness, (ii) anatomic complexity, (iii) feasibility with existing technology, (iv) risk of procedure, (v) risk of not undergoing the procedure, (vi) urgency of procedure, (vii) availability of procedure in under-resourced areas, (viii) morbidity of converting to a nonautonomous procedure, and (ix) anesthetic requirements. An ideal procedure would be common, requiring simple anatomical dissection, feasible with existing technology, have low risk, have high risk if not performed acutely, have limited access in under-resourced areas, have low morbidity for converting to a nonautonomous procedure (e.g., it is not required to open a major blood vessel that cannot be closed), and have a simple anesthetic solution (e.g., regional anesthesia versus cardiac anesthesia with heart bypass).

Considering these factors, we propose that the most feasible first deployment of autonomous surgery would be the initial assessment and surgical treatment of patients following high-energy trauma. We envision this instrument as a modified operating table with an embedded CT scanner and multiple robotic arms, with deployment in active military or rural civilian settings. This model would feature real-time ML interpretation of a whole-body CT scan that would identify long-bone and pelvic trauma. AI would then offer an immediate surgical plan, such as standard femoral and tibial intramedullary titanium rods or external fixators, for stabilizing major fractures to prepare the patient for triage.

Implementing this approach will involve modifying existing surgical tools to accommodate robotic use, identifying physical constraints on tissue manipulation to avoid patient injury, monitoring blood flow and nerve function to the distal extremities during the procedure, and developing maneuvers to work within these constraints to reconstruct patient’s anatomy to approximate their preinjury state. To accomplish this last goal, the AI engine will eventually be superior to human surgeons because it will be able to reference the patient’s contralateral, likely uninjured, anatomy, use anatomical reference texts from other patients with similar anatomy, and potentially render a 3D reconstruction of the fractured bone.

Generative models represent a substantial advancement in AI-driven orthopedic robotic surgery, enabling precise image reconstruction and autonomous surgical guidance. Recent breakthroughs in large language models and visual-language models allow foundation models to analyze radiological images alongside textual reports and clinical notes. This capability enhances the detection of subtle anatomical features while also enabling high-fidelity 3D reconstruction and localization. While today most of intraoperative image guided orthopedic surgeries involve a preoperative CT scan merged with intraoperative x-ray, there are notable limitations in poor registration accuracy and patient shift.

With generative models, one of the most obvious use cases is the generation of 3D reconstruction from 2D imaging directly from 2D x-ray, when 3D features projected into the 2D plan present an unrecoverable 3D localization of anatomy necessary. Without the use of volumetric imaging such as CT, this inverse problem of 2D to 3D is unsolvable. However, the use of generative models, where 2D-to-3D translation has been learned and encoded, provides the foundation for converting 2D to 3D images autonomously and accurately because the 2D-to-3D anatomy translation is now explicitly encoded in the large network model covering a spectrum of anatomy from previous patient cases. Not only does this allow for 2D-to-3D reconstruction natively without volumetric imaging, but it can ultimately be interpreted by tracked instruments to carry out procedures autonomously. When married with robotics, generative models for imaging and for 3D reconstruction can be leveraged for steering instruments in tasks such as bone debridement or implant alignment and may be done in an optimized fashion by allowing intermittent imaging and robotic steering to work in unison. This provides intraoperative guidance with minimized irradiation to the patient and the physicians.

#### The orthopedic surgeon’s perspective

Orthopedic surgery often involves replacing portions of human bones with implants. The success of the surgery procedures largely depends on the surgery planning process and osseointegration result of the implants, ensuring their stability and long-term functionality. A notable challenge in orthopedic surgery lies in the longevity and biocompatibility of bone implants (*165*, *166*). Joint replacements and other orthopedic implants are prone to wear over time, potentially necessitating revision surgeries. The materials used in these implants may also lead to issues such as implant rejection or bone infections, particularly in elderly patients or those with conditions like diabetes. Advances in bone implants and their surgical planning, especially those with integrated drug delivery systems (*167*) and sensors (*168*), are revolutionizing orthopedic treatments with real-time monitoring and targeted therapy.

Drug delivery systems integrated in bone implants allows for the controlled release of drugs over time after the surgery, promoting bone growth (osteogenesis) during surgery recovery. The implants are often coated with biocompatible polymers such as polylactic acid, polycaprolactone, or poly-lactic-*co*-glycolic acid, which can be loaded with antibiotics, anti-inflammatory agents, or other drugs (*169*). Additionally, microporous and nanoporous coatings can be customized to release drugs at specific rates, further enhancing therapeutic outcomes. Hydrogel-based systems offer another method for drug delivery, where hydrogels can be incorporated into the device (*168*, *169*) or applied as a coating (*170*, *171*). These systems form a matrix that allows for the controlled release of therapeutic agents, making them particularly effective for delivering growth factors, antibiotics, or anti-inflammatory drugs directly to the implant site. As one example, antibiotic-releasing implants (*172*) play a critical role in infection control, especially for high-risk patients undergoing surgery. Implants designed to release bone growth factors are used to enhance osteogenesis, thereby improving the success rates of bone healing and integration (*173*).

Smart implants embedded with sensors represent a substantial advancement in the ability to monitor and optimize the surgery performance in a long term (*174*). Technologies such as pressure and load sensors, including resistive (*175*) and capacitive sensors (*176*), generate electrical signals in response to mechanical stress, enabling continuous monitoring of the implant’s load-bearing capacity and the early detection of potential failures (*177*, *178*). For example, the OrthoSensor Knee Implant (*179*) is equipped with sensors that measure joint pressure and load during surgery, ensuring proper alignment and fit, thereby improving surgical outcomes. Similarly, smart hip implants (*180*) are being designed with sensors that monitor load distribution and detect loosening or failure over time facilitating long-term planning of surgery and intervention.

Looking ahead, the field of bone implants continues to grapple with notable challenges. A critical issue is surgery planning and osseointegration as implants must withstand substantial forces, particularly in load-bearing bones, without succumbing to wear, corrosion, or fatigue over time. Achieving this requires smart bone implants capable of delivering stem cells and therapeutic agents on-demand, over extended periods. Furthermore, continuous, long-term monitoring of bone conditions is essential for closed-loop, on-demand surgical functions. The integration of wireless communication and power technologies into bone implants is demanded. Methods like inductive coupling and wireless power transfer enable the wireless powering of sensors embedded within implants (*181*). These sensors can communicate using technologies such as radio frequency and Bluetooth low energy (*182*, *183*), although miniaturization and long-term stability remain key considerations. Last, the need for precise customization and fit presents additional hurdles due to the variability in individual bone structures and the high costs of patient-specific solutions. These challenges underscore the need for ongoing innovation in materials science, surgical planning, and postoperative care to improve the efficacy and longevity of bone implants.

### Microrobotics for inaccessible surgery and therapy

#### Current landscape and near-term translation opportunities

**Fig. 4.**
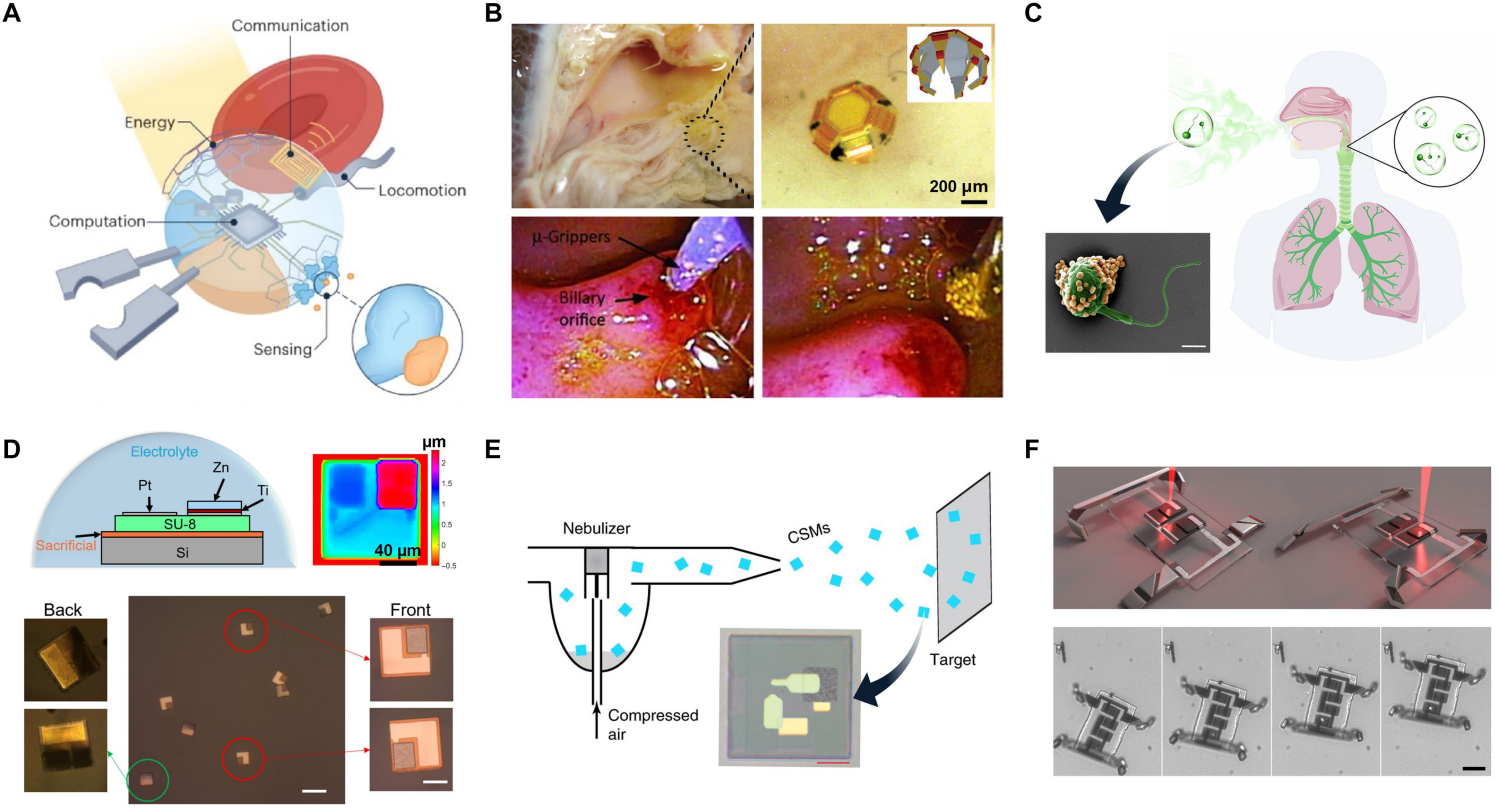
Recent developments in microrobotics for surgery and biomedical applications. (**A**) Conceptual schematics of micrometer-scale autonomous microrobots. Reproduced from (*185*). (**B**) Untethered microgrippers for biopsy of a porcine liver (top), and delivery and retrieval of microgrippers through a magnetic catheter into the porcine biliary orifice (bottom). Reproduced from (*206*) with permission from John Wiley & Sons. 2012 Wiley. (**C**) Schematic of an algae-based biohybrid microrobot nebulizer platform for inhalable lung delivery, and a scanning electron microscopy image of a drug-loaded algae robot. Scale bar (inset), 500 nm. Adapted from (*199*), licensed under the Creative Commons CC BY license. (**D**) Schematic and height profile of the Zn-air microbattery (top), and micrographs of the released microbattery (bottom). Scale bar, 50 μm. Reproduced from (*200*), G. Zhang *et al.*, *Science Robotics*, DOI: 10.1126/scirobotics.ade4642 (2024), AAAS. (**E**) Schematic and micrograph of aerosolizable microrobots, also referred to as colloidal state machines (CSMs), including components of a photodiode, chemiresistor, and memory. Scale bar, 25 μm. Reproduced from (*201*). (**F**) Schematic and micrograph of electronically integrated microrobots with photodiodes and actuators. Scale bar, 20 μm. Reproduced from (*202*).

Microrobotics in medicine often refers to surgical and medical devices ranging from the centimeter scale down to the nanometer scale (*184*, *185*). Although these platforms vary widely in manufacturing processes, physical properties, and functionality across different size ranges, they share a common goal of enabling surgical or interventional tasks in regions that are inaccessible to conventional instruments. As the overall size decreases, these devices can access smaller anatomical pathways or provide highly localized therapies; however, they also face greater constraints on power, actuation, sensing, and computational capacity (*185*–*188*). At the centimeter and millimeter scales, microrobots may function as specialized end effectors on larger robotic systems. At the micrometer scale, multiagent swarms may be required to perform tasks beyond the capacity of a single device. At the nanoscale, the distinction between microrobotic devices and targeted medication delivery becomes less clear. Although many current microrobotic systems rely on external power or control, fully autonomous operation will require greater integration of key components such as locomotion, sensing, computation, energy storage, and communication (Fig. 4A) (*185*).

Devices with dimensions in the centimeter-to-millimeter range can serve as a bridge between conventional macroscale surgical robots and microscale platforms. Their dimensions allow for a level of structural robustness and the inclusion of actuators, sensors, or limited power storage, enabling them to interact with macroscopic tissue. Examples include ingestible capsules for imaging and drug delivery in the GI tract (*52*, *53*, *58*), catheter-based microrobots for clot removal (*42*, *43*), and specialized end effectors for minimally invasive procedures (*189*, *190*). Advanced additive or laminate-based processes are frequently used to manufacture these millimeter-scale devices, offering complex multilayer geometries with mechanical properties that approach those of bulk materials (*191*, *192*). Rapid prototyping and short fabrication cycles make it feasible to develop application-specific tools that reduce reliance on large external robots, shifting complexity inside the patient in a controlled manner (*193*).

Below the millimeter range, power and control constraints become more severe as most micrometer-scale systems lack onboard batteries or high-level processors and thus rely on externally supplied power for locomotion or task execution (*185*). One example is untethered microgrippers, which can be deployed under magnetic guidance for tissue biopsy. These microgrippers harness energy from differential residual stress in thin films, combining chromium (in tension) and gold (in neutral-stress bilayers), environmentally triggerable materials (e.g., low-temperature wax and gels) or drug patches to enable biopsy and bioresident devices (theragrippers) (*74*, *194*). Because they do not require conventional batteries, they can be miniaturized to the extent of capturing red blood cells (*195*). These microgrippers have already been tested in preclinical animal models (Fig. 4B). Polymeric microgrippers and theragrippers further improve biocompatibility and allow reversible actuation (*196*, *197*). By integrating vision-based or CT-based modalities, soft microgrippers loaded with nanoparticles for contrast or magnetic functionality can be used for a variety of tasks, including pick-and-place and haptic control. However, robotic demonstrations using untethered microgrippers have been carried out in a dish or in vitro models (*198*) and translation to in vivo practice has yet to be achieved.

Additionally, Wang and co-workers developed inhalable biohybrid microrobots that can be delivered noninvasively to the lung via an aerosol-dispensing nebulizer (Fig. 4C) (*199*). In this system, algae serve as the primary body of the microrobots and are functionalized with platelet membrane–coated polymeric nanoparticles. A key advantage of micrometer-scale platforms is their potential for deployment through extremely small catheters, aerosol sprays, or ingestible vehicles, thereby minimizing disruption to surrounding tissues. Nevertheless, limited force output and the complexity of coordinating large numbers of devices remain substantial challenges. Despite these advances, most microrobotic systems remain externally powered and supervised, which constrains their achievable autonomy. This motivates the integration of onboard power and computation together with verifiable safety, tracking, and retrieval strategies.

#### Future directions: Toward untethered autonomous microrobotic surgery

Achieving fully autonomous microrobotic surgery involves more than device miniaturization. Energy storage remains a major hurdle as standard battery technologies cannot be readily scaled down to micrometer dimensions, and recharging a large number of microrobots in situ is difficult. Preliminary developments in miniature batteries, such as the Zn-air microbattery shown in Fig. 4D, illustrate potential solutions for local power storage (*200*). Other major challenges include developing fabrication processes, implementing onboard computation, and achieving efficient mass-manufacturing. Koman *et al.* (*201*) introduced aerosolizable microrobots that integrate photodiode, chemical sensor, and memristor-based memory, thereby demonstrating the ability to record environmental chemical data onboard (Fig. 4E). Miskin *et al.* (*202*) further demonstrated lithographically fabricated, electronically integrated microrobots capable of walking at sub-hundred-micrometer scales (Fig. 4F). While these achievements mark important milestones, these technologies remain at a research stage and are still far from being applicable to fully autonomous μ-RAS platforms.

Several safety concerns must be addressed before microrobots can be deployed as μ-RAS tools. One key concern is operational safety. A fully autonomous microrobot must sense and adapt to the environment without continuous human intervention and thus requires robust fail-safes. Most systems rely on external imaging or high-level control to reduce onboard complexity and mitigate the risks associated with unsupervised decision-making among large numbers of small agents (*203*). Hybrid strategies, in which microrobots incorporate simple local control loops while supervised by an external system with more comprehensive capabilities, offer a practical near-term solution.

Another major safety concern relates to biocompatibility and the challenges of control and retrieval at micrometer scales. Larger, millimeter-scale devices can often be removed endoscopically or pass naturally through bodily lumens, but tracking and recovering swarms of micrometer-scale robots is more complex. This difficulty has prompted the development of biodegradable or bioresorbable designs, which minimize long-term risks by degrading safely within the body over a defined period (*204*, *205*).

While fully autonomous microrobotic surgery remains an aspirational goal, advances in power systems, materials, and control algorithms continue to expand the clinical potential of these devices. In the near term, millimeter-scale platforms are likely to see increased use in endoluminal or minimally invasive interventions, relying partly on teleoperation or external fields for power. By contrast, micrometer-scale robots hold promise for tasks such as localized therapy, biopsies in confined spaces, or slow, low-force interventions over extended intervals. Future developments may include robust wireless power transmission, reliable onboard energy storage, and increasingly sophisticated AI-driven control. Overcoming the remaining engineering and regulatory challenges will be critical to fulfilling the promise of microrobotics in safely extending surgical capabilities to previously inaccessible or high-risk regions of the human body.

## DISCUSSION

### Outlook

The authors have the view that autonomy in RAS and μ-RAS should be introduced where it most clearly reduces surgeon burden and case-to-case variability, beginning with assistive functions rather than full automation. Across our five domains, clinicians value capabilities that lower cognitive load and ergonomic strain while improving consistency in difficult subtasks. This includes stable visualization and camera control, route planning within constrained anatomy, guarded instrument guidance near critical structures, and routine but attention-heavy chores (retraction, suction, device positioning, and lesion targeting). The near-term goal differs by field: vascular navigation for thrombectomy and PCI, route following and lesion localization in endoscopy, force-limited tissue handling in laparoscopy, OCT-guided micromanipulation in ophthalmology, and fluoroscopy-linked alignment in orthopedics. Still, the overall principle is the same, to automate the narrow, high-value steps and improve safety, speed, or access.

Clinical value must be shown where it matters to patients and services, including time-to-therapy in emergencies (e.g., thrombectomy), completeness, and accuracy of targeted actions (biopsy yield, ablation continuity, and implant alignment), complication rates linked to tissue handling, and surgeon musculoskeletal injury. Economic viability follows from shorter case times, fewer disposables and less radiation, and a shorter learning curve that brings low-volume centers closer to high-volume performance. For μ-RAS, practicality means dependable localization, retrieval or biodegradation, and control strategies that fit through existing scopes and catheters. The choice between tethered and untethered should be driven by the clinical needs.

Across our five domains, progress toward task-specific autonomy will depend on integrating sensing and data streams already used in current robotic workflows. Endoluminal navigation is driven primarily by endoscopic video, while endovascular and orthopedic procedures are guided by fluoroscopy and CT-based navigation. In laparoscopy, coregistered visual inputs including fluorescence imaging and laser speckle imaging can be presented as AR overlays, and, in ophthalmology, real-time fusion of OCT and digital microscope images together with a digital twin illustrates tight integration of imaging and actuation for micromanipulation. AI- and ML-based methods can help translate these multimodal inputs into actionable guidance, complemented by positional tracking, force sensing, and haptic feedback to limit tissue loading and by monitoring tissue-specific physiological signals where applicable. In addition, multifunctional devices that couple diagnosis and therapy, such as ingestible smart pills or implants that integrate sensing with drug delivery, may extend these benefits beyond the immediate procedure.

A realistic roadmap starts with low-level, task-specific autonomy applied to targeted subtasks, supported by rigorous, specialty-specific metrics, and staged trials that keep the surgeon firmly in the loop. Establishing data standards that integrate imaging, video, kinematics, forces, and outcomes will accelerate generalization and external review, while responsibility should be aligned with the same task boundaries already used in surgical training and quality assurance. Building on advances in imaging and control, the path toward fully autonomous RAS and μ-RAS will require not only algorithmic progress but also durable hardware, reliable sensors, and protocols that can accommodate the variability of real-world clinical conditions. Regulatory and professional oversight will determine how and when these systems transition from laboratory prototypes to mainstream surgical tools. Although early demonstrations suggest the potential to reduce complications and improve efficiency, rigorous validation is essential to confirm consistent performance, safety, and clinical benefit. With deliberate collaboration among engineers, clinicians, and policymakers, autonomous RAS and μ-RAS systems can ultimately progress from experimental concepts to dependable elements of surgical practice, delivering precise, high-quality care that is both effective and widely acceptable.
